# Robotic versus laparoscopic adrenalectomy: five-year comparative outcomes from a high-volume tertiary endocrine surgery center

**DOI:** 10.1007/s11701-025-03133-3

**Published:** 2026-01-10

**Authors:** Sezer Akbulut, Tugba Matlim Ozel, Aykut Celik, Gorkem Yildiz, Sebnem Burhan, Emrecan Deniz, Serkan Sari

**Affiliations:** 1 Department of General Surgery, Division of Endocrine Surgery, University of Health Sciences Turkey, Basaksehir Cam and Sakura City Hospital, Istanbul, Turkey; 2 Department of Endocrinology, University of Health Sciences Turkey, Basaksehir Cam and Sakura City Hospital, Istanbul, Turkey

**Keywords:** Adrenal gland surgery, Robotic adrenalectomy, Robotic surgery, Laparoscopic adrenalectomy, Minimally invasive surgery

## Abstract

Laparoscopic adrenalectomy (LA) is the standard minimally invasive approach, whereas robotic adrenalectomy (RA) is increasingly adopted for its ergonomic and technical advantages. Whether these benefits improve perioperative outcomes—particularly by adrenal laterality—remains unclear. This study compared RA and LA outcomes via structured side-specific analysis. A total of 198 patients were screened in this retrospective cohort study, which included adults who underwent minimally invasive adrenalectomy between June 2020 and September 2025. Patients with paragangliomas, recurrent disease, or open adrenalectomy were excluded. Clinical, operative, and postoperative variables were collected, and laterality-specific subgroup analyses and multivariable linear regression were performed. A total of 181 patients were analyzed (126 LA, 55 RA). The length of hospital stay was significantly shorter in the RA group (*p* = 0.019), whereas the operative time was significantly longer in the RA group than the LA group (*p* < 0.001). No significant differences were observed between techniques regarding complications, transfusions, or conversion rates (all *p* > 0.05). When stratified by laterality, the RA consistently demonstrated longer operative times for both right- and left-sided procedures (*p* = 0.001 and *p* < 0.001, respectively). In the multivariate analysis, only the surgical approach and tumor diameter independently affected the operative time (both *p* < 0.001). Robotic adrenalectomy demonstrated perioperative safety comparable to that of laparoscopy while providing the advantage of a shorter hospital stay despite longer operative times. Given its similar complication and conversion profiles, RA represents a feasible and ergonomically favorable procedure in endocrine surgery centers.

## Introduction

Laparoscopic adrenalectomy (LA), first introduced by Gagner et al. in 1992, revolutionized the surgical management of adrenal tumors [1]. Over the past three decades, this minimally invasive approach has gained global acceptance among endocrine surgeons and is now considered the gold standard for most adrenal pathologies [2, 3]. Despite its proven safety and efficacy, LA has inherent technical limitations. Conventional laparoscopy offers only two-dimensional imaging, uses rigid instruments with limited mobility, and relies on an assistant-controlled camera system that may compromise stability and precision. These factors can impair depth perception and maneuverability, increasing surgeon fatigue during complex dissections in the deep retroperitoneal space. Although modern three-dimensional high-definition systems have improved visualization, laparoscopic technology remains restricted by the use of straight instruments, hand tremors, and nonintuitive hand–eye coordination. The robotic platform was subsequently developed to overcome these inherent shortcomings of conventional laparoscopy [4, 5].

The development of robotic surgery accelerated following the approval of the da Vinci Surgical System by the U.S. Food and Drug Administration. Soon after, Horgan and Vanuno reported the first clinical series of robotic-assisted adrenalectomy in the United States. Since then, the robotic platform has been progressively integrated into endocrine surgery as an advanced minimally invasive technology designed to overcome the technical and ergonomic limitations of conventional laparoscopy through enhanced visualization, improved precision, and greater instrument dexterity [6].

Robotic adrenalectomy (RA) has increasingly been embraced in high-volume tertiary endocrine surgery centers as a viable alternative to LA. Numerous comparative studies have explored whether the technological and ergonomic advantages of the robotic platform translate into measurable clinical benefits. Despite this growing interest, its widespread global adoption is still constrained by high capital and maintenance costs, limited system availability, and persistent debate regarding its cost-effectiveness relative to conventional laparoscopy [7, 8]. An increasing body of evidence continues to explore the potential benefits of RA over LA, with many surgeons recognizing the robotic system as a valuable and ergonomically favorable alternative, particularly in complex adrenal procedures [9, 10].

This study aimed to compare the intraoperative and postoperative outcomes of robotic and laparoscopic adrenalectomy performed at our high-volume endocrine surgery center over a five-year period. The analysis was designed to assess the safety, clinical performance, and potential advantages of the robotic approach within contemporary minimally invasive adrenal surgery.

## Materials and methods

This retrospective single-center cohort study was conducted at the Department of General Surgery, Division of Endocrine Surgery, Basaksehir Cam and Sakura City Hospital, University of Health Sciences, Istanbul, Turkey. All patients evaluated by a multidisciplinary team consisting of endocrinologists, radiologists, and endocrine surgeons who underwent adrenal surgery between June 2020 and September 2025 were reviewed. A total of 198 patients were screened. Patients aged ≥ 18 years who underwent minimally invasive adrenalectomy—either laparoscopic (LA) or robotic (RA)—were eligible for inclusion. The exclusion criteria were paraganglioma (*n* = 6), recurrent adrenal tumors requiring reoperation (*n* = 7), and primary open adrenalectomy (*n* = 6). After these patients were excluded, the remaining patients composed the study cohort for comparative analysis. Baseline demographic and clinical variables—including age, sex, body mass index (BMI), ASA score, previous abdominal surgery, and preoperative diagnosis—were collected from the institutional electronic database. Intraoperative parameters included operative time, conversion to laparoscopy or open surgery, and intraoperative complications. The postoperative outcomes assessed were length of hospital stay, need for blood transfusion, postoperative complications, pathological findings, and 30-day mortality. The study was approved by the Institutional Review Board of Basaksehir Cam & Sakura City Hospital (Approval No: 2025- 66).

### Surgical procedure

#### Laparoscopic transabdominal approach

Patients were placed in the lateral decubitus position with table flexion to increase the distance between the costal margin and the iliac crest. The first trocar was inserted 2 cm below the costal margin along the anterior axillary line via an optical entry technique. On the right side, three additional trocars were placed along the subcostal line. On the left side, two trocars were inserted after the camera port, with a third port added when exposure was limited. This configuration provides clear visualization of the adrenal gland and surrounding structures [11].

### Robotic transabdominal approach

**Fig. 1:**
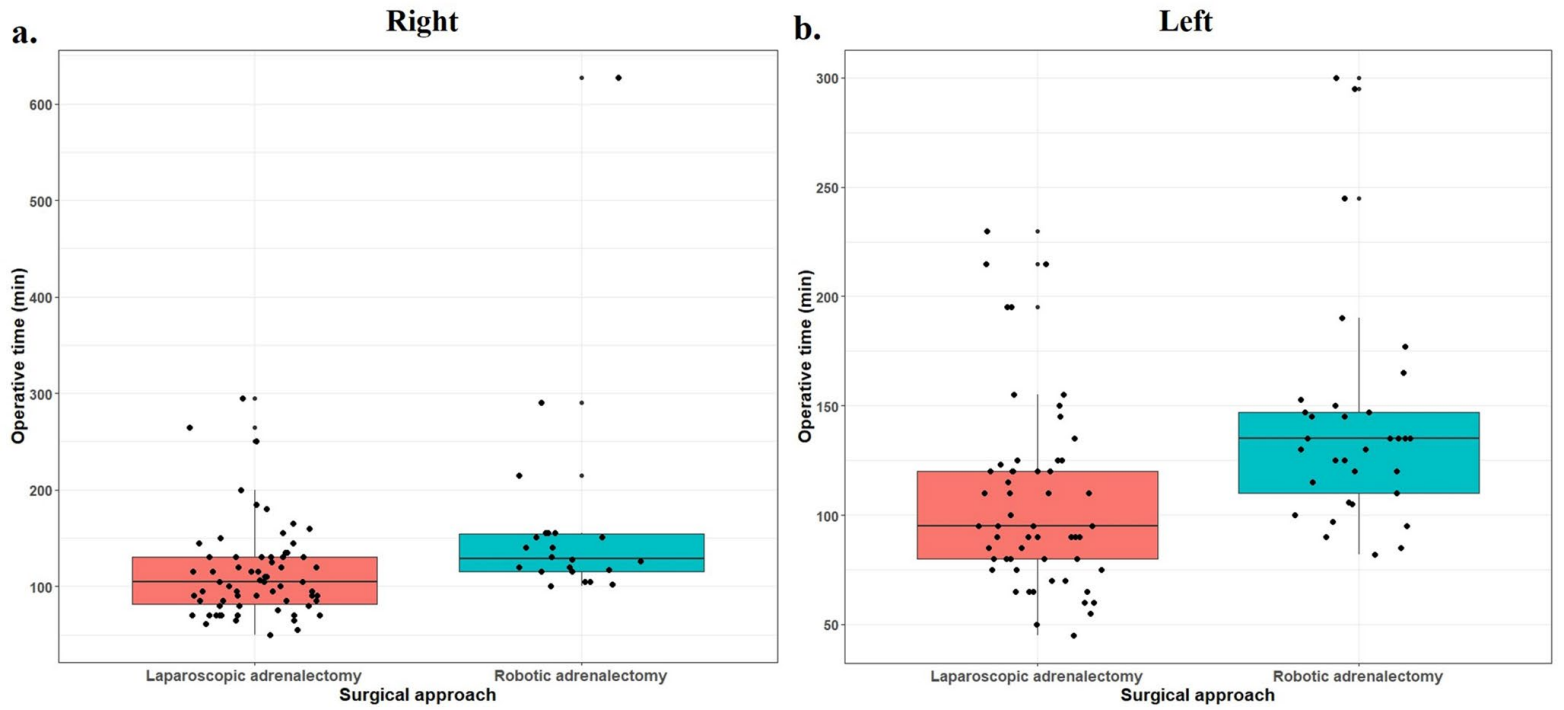
Boxplot illustrating the distribution of operative times between Laparoscopic adrenalectomy and Robotic adrenalectomy groups for right- and left-sided procedures

Robotic lateral transabdominal adrenalectomy was performed via the da Vinci Xi system. Trocar placement mirrored the laparoscopic configuration, with an additional assistant port in the ipsilateral lower quadrant. Maryland bipolar dissector were introduced through the medial port and monopolar scissors were introduced through the lateral trocars. Dissection and hemostasis were performed via a Maryland bipolar dissector and monopolar scissors. The adrenal vein was identified and clipped under direct vision (Fig. 1).

### Retroperitoneal approach (laparoscopic and robotic)

For the retroperitoneal approach, three ports were placed obliquely beneath the costal margin. The camera port was inserted midway between the medial and lateral landmarks, just lateral to the paraspinous muscles. After a 1.5-cm skin incision, the retroperitoneal space was entered via Metzenbaum scissors and expanded with gentle blunt dissection. The medial port was placed 2–3 cm below the inferior margin of the 12th rib and angled slightly inferiorly to stay within the retroperitoneal plane. The lateral port was positioned below the costal margin to optimize triangulation. A 10-mm balloon-tipped trocar was used at the camera port to maintain the retroperitoneal space [12]. The same configuration was used for robotic retroperitoneal adrenalectomy, with an additional assistant trocar for suction and retraction. Dissection and hemostasis were performed mainly with a Maryland bipolar dissector and monopolar scissors, and the adrenal vein was clipped under direct visualization.

### Operative standardization

All procedures were video-recorded. Robotic adrenalectomies were performed by the senior endocrine surgeon (S.S.) to ensure technical consistency, accompanied by an endocrine surgery board-certified general surgeon serving as the assistant surgeon. The robotic platform was installed in a dedicated operating room, eliminating setup delays. The operative time was measured from skin incision to closure. All patients received antibiotic and antithrombotic prophylaxis according to the institutional protocol, and a surgical drain was routinely placed.

### Statistical analysis

The data were analyzed using the SPSS version 25.0 software (IBM Corp., Armonk, NY, USA). Within the scope of descriptive statistics, categorical variables are presented as numbers (n) and percentages (%), whereas continuous variables are reported as the mean ± standard deviation (SD) or median and interquartile range (Q1–Q3), depending on their distributional characteristics. For comparisons of categorical variables between groups, Pearson’s chi-square test or Fisher’s exact test was used, based on the observed frequency distributions. The distributional properties of continuous variables were evaluated statistically using the Kolmogorov–Smirnov and Shapiro–Wilk tests, according to sample size, and visually through Q–Q plots and histogram inspection. Homogeneity of variances was assessed with Levene’s test. For continuous variables with a normal distribution, comparisons between two independent groups were performed using the independent samples t-test, whereas the Mann–Whitney U test was applied when the normality assumption was not met. To determine the independent factors influencing operative time, a multivariable linear regression analysis was conducted, and the results were reported with β coefficients and 95% confidence intervals. All statistical analyses were performed in a two-tailed manner, and a p-value < 0.05 was considered to indicate statistical significance.

## Results

A total of 181 patients were included in the final analysis, of whom 126 (69.6%) underwent LA and 55 (30.4%) underwent RA. Among the LA group, 7 patients were treated via the retroperitoneal laparoscopic approach, whereas the remaining procedures were performed transabdominally. In the RA group, 1 patient underwent retroperitoneal robotic adrenalectomy, and all other robotic procedures were performed via the transabdominal approach. The mean age was 50.33 ± 11.78 years (range, 21–76) and the mean BMI was 29.85 ± 5.96 kg/m². Female patients constituted 69.6% of the cohort.

Baseline characteristics were comparable between groups with respect to age (*p* = 0.732), sex (*p* = 0.547), ASA class (*p* = 0.235), and history of previous abdominal surgery (*p* = 0.725). BMI was significantly greater in the RA group (31.68 ± 6.39 vs. 29.05 ± 5.60; *p* = 0.006). The tumor diameter was greater in the LA group (4.5 cm vs. 3.8 cm; *p* = 0.039). The laterality and preoperative diagnosis distributions did not differ significantly between the groups (*p* = 0.132 and *p* = 0.367, respectively). Three patients underwent bilateral adrenalectomy, resulting in one patient with dual pathology. The most common diagnoses in the LA group were Cushing adenoma (38.1%), Conn adenoma (19.8%), and pheochromocytoma (19.0%), whereas in the RA group, Cushing adenoma (49.1%), Conn adenoma (21.8%), and pheochromocytoma (12.7%) were most frequently observed (Table 1).

**Table 1:** Baseline characteristics of patients undergoing laparoscopic vs. Robotic adrenalectomy

		LA (*n* = 126)	RA (*n* = 55)	*P* values
Gender	Female	86 (68.3%)	40 (72.7%)	0.547^a^
	Male	40 (31.7%)	15 (27.3%)	
Age (years)	mean ± SD	50.13 ± 11.66	50.78 ± 12.13	0.732^c^
BMI (Kg/m²)	mean ± SD	29.05 ± 5.6	31.68 ± 6.39	**0.006** ^**c**^
ASA	I	1 (0.8%)	1 (1.8%)	0.235^b^
	II	74 (58.7%)	25 (45.5%)	
	III	50 (39.7%)	29 (52.7%)	
	IV	1 (0.8%)	0 (0%)	
Previous abdominal surgery	No	119 (94.4%)	53 (96.4%)	0.725^b^
	Yes	7 (5.6%)	2 (3.6%)	
Pathologic lesion diameter (cm)		4.5 (3–6.5)	3.8 (2.7–5.2)	**0.039** ^**d**^
Laterality	Right	66 (52.4%)	22 (40%)	0.132^b^
	Left	57 (45.2%)	33 (60%)	
	Bilateral	3 (2.4%)	0 (0%)	
Preoperative diagnosis	Cushing adenoma	48 (38.1%)	27 (49.1%)	0.367^b^
	Conn adenoma	25 (19.8%)	12 (21.8%)	
	Pheochromocytoma	24 (19.0%)	7 (12.7%)	
	Metastasis	12 (9.5%)	1 (1.8%)	
	ACC	1 (0.8%)	0 (0.0%)	
	NFA	15 (11.9%)	8 (14.5%)	
	Dual pathology	1 (0.8%)	0 (0.0%)	

^a^Chi-square test with n (%)

^b^Fisher exact test with n (%)

^c^Student’s t-test with mean ± SD

^d^Mann Whitney U test with median (Q1, Q3)

LA: Laparoscopic adrenalectomy, RA: Robotic adrenalectomy, BMI: Body mass index (kg/m^2^), ASA: American Society of Anesthesiologists, ACC: Adrenocortical carcinoma, NFA: Nonfunctioning adrenal adenoma

Dual pathology = Cushing and Pheochromocytoma

Bold values indicate statistically significant differences (*p* 0.05).

The perioperative outcomes revealed that the length of hospital stay was significantly shorter in the RA group (3 [2, 3] days vs. 3 [3, 4] days; *p* = 0.019), whereas the operative time was significantly longer (130 [115–151] vs. 100 [80–130] minutes; *p* < 0.001). Conversion to open surgery occurred in 3.3% (*n* = 6) of patients, and conversion from RA to laparoscopy occurred in 0.6% (*n* = 1) of patients. There were no postoperative deaths within 30 days of surgery. The conversion rates, transfusion requirements, intraoperative complications, postoperative complications, and pathological diagnosis distributions were similar between the groups (all *p* > 0.05) (Table 2). Conversion to open or laparoscopic surgery was required for several intraoperative reasons. In one patient in whom a robotic transabdominal approach was initially attempted, conversion to retroperitoneal laparoscopic surgery was performed because of inadequate visualization related to hepatomegaly. Among patients undergoing robotic adrenalectomy, conversion to open surgery was necessary due to bleeding from the adrenal mass in one patient and technically challenging exploration associated with posterior tumor extension toward the inferior vena cava in another. In patients undergoing laparoscopic adrenalectomy, conversion to open surgery was required because of left renal vein injury related to tumor extension into the renal hilum, bleeding from the adrenal mass, liver injury secondary to hepatomegaly, or dense adhesions between the adrenal mass and the diaphragm precluding safe dissection. Intraoperative complications in the LA group included splenic injury in two patients, liver injury in one patient, and left renal vein injury in one patient, whereas RA was associated with liver injury in one patient. Postoperative complications in the LA group included intra-abdominal abscess, pulmonary embolism, and myocardial infarction. In the RA group, the postoperative complications included one incarcerated incisional hernia and one patient who required re-exploration due to a postoperative hematoma.

**Table 2:** Perioperative and postoperative characteristics of RA vs. LA

		LA (*n* = 126)	RA (*n* = 55)	*P* values
Length of hospital stay (days)		3 (3–4)	3 (2–3)	**0.019** ^**d**^
Operative time (min)		100 (80–130)	130 (115–151)	**< 0.001** ^**d**^
Surgical Conversion	No	122 (96.8%)	52 (94.5%)	0.429^b^
	Conversion to open surgery	4 (3.2%)	2 (3.6%)	
	Conversion to laparoscopy	0 (0.0%)	1 (1.8%)	
Blood transfusions	No	120 (95.2%)	51 (92.7%)	0.495^b^
	Yes	6 (4.8%)	4 (7.3%)	
Postoperative 30-day mortality		0	0	**-**
Complications	No	119 (94.4%)	52 (94.5%)	1.000^b^
	Intraoperative complication	4 (3.2%)	1 (1.8%)	
	Postoperative complication	3 (2.4%)	2 (3.7%)	
Pathological diagnosis	Adrenocortical adenoma	76 (60.3%)	40 (72.7%)	0.155^b^
	Pheochromocytoma	24 (19.0%)	5 (9.1%)	
	ACC	4 (3.2%)	5 (9.1%)	
	Metastasis	12 (9.5%)	1 (1.8%)	
	Myelolipoma	3 (2.4%)	2 (3.6%)	
	Lymphangioma	1 (0.8%)	1 (1.8%)	
	Adrenal cystic lesion	2 (1.6%)	0 (0.0%)	
	Ganglioneuroma	1 (0.8%)	0 (0.0%)	
	Leiomyosarcoma	1 (0.8%)	0 (0.0%)	
	Schwannoma	1 (0.8%)	1 (1.8%)	
	Dual pathology	1 (0.8%)	0 (0.0%)	

^b^Fisher exact test with n (%)

^d^Mann Whitney U test with median (Q1, Q3)

LA: Laparoscopic adrenalectomy, RA: robotic adrenalectomy, ACC: Adrenocortical carcinoma,

Dual pathology = Adrenocortical Adenoma + Pheochromocytoma

Bold values indicate statistically significant differences (*p* 0.05).

According to the laterality-based analyses, after three patients who underwent bilateral adrenalectomy were excluded, the operative times were significantly longer in the RA group for both right-sided (*p* = 0.001) and left-sided procedures (*p* < 0.001).Fig (1) The length of hospital stay was shorter for RAs on the left side (*p* = 0.023), whereas no difference was observed on the right side (*p* = 0.385). The complication and transfusion rates were comparable between the two sides (Table 3).

**Table 3:** Comparison of sociodemographic and clinical variables between RA and LA according to laterality

		Right side (*n* = 88)			Left side (*n* = 90)		
		LA (*n* = 66)	RA (*n* = 22)	*P* values	LA (*n* = 57)	RA (*n* = 33)	*P* values
Gender	Female	41 (62.1%)	19 (86.4%)	**0.034** ^**a**^	42 (73.7%)	21 (63.6%)	0.316^a^
	Male	25 (37.9%)	3 (13.6%)		15 (26.3%)	12 (36.4%)	
Age (years)		49.64 ± 10.92	47.73 ± 12.33	0.494^c^	50.70 ± 12.14	52.82 ± 11.74	0.422^c^
BMI (Kg/m²)	< 30	42 (63.6%)	13 (59.1%)	0.703^a^	28 (49.1%)	15 (45.5%)	0.737^a^
	≥ 30	24 (36.4%)	9 (40.9%)		29 (50.9%)	18 (54.5%)	
Previous abdominal surgery	No	62 (93.9%)	21 (95.5%)	1.000^b^	54 (94.7%)	32 (97%)	1.000^b^
	Yes	4 (6.1%)	1 (4.5%)		3 (5.3%)	1 (3%)	
Operative time (min)		105 (80–130)	129 (115–155)	**0.001** ^**d**^	95 (77.5–121.5)	135 (108–148.5)	**< 0.001** ^**d**^
Complications	No	62 (93.9%)	19 (86.3%)	0.590^b^	55 (96.5%)	33 (100%)	0.530^b^
	Intraoperative complication	1 (1.5%)	1 (4.5%)		2 (3.5%)	0 (0%)	
	Postoperative complication	3 (4.5%)	2 (9%)		0 (0%)	0 (0%)	
Blood transfusions	No	63 (95.5%)	20 (90.9%)	0.595^b^	54 (94.7%)	31 (93.9%)	1.000^b^
	Yes	3 (4.5%)	2 (9.1%)		3 (5.3%)	2 (6.1%)	
Length of hospital stay (days)		3 (3–4)	3 (2–4.25.25)	0.385^d^	3 (3–4)	3 (2–3)	**0.023** ^**d**^

^a^Chi-square test with n (%)

^b^Fisher exact test with n (%)

^c^Student’s t-test with mean ± SD

^d^Mann Whitney U test with median (Q1, Q3)

LA: Laparoscopic adrenalectomy, RA: Robotic adrenalectomy, BMI: Body mass index (kg/m^2^)

Bold values indicate statistically significant differences (*p* 0.05).

Multivariate linear regression revealed that the surgical approach was an independent predictor of the operative time. Compared with LA, RA was associated with an additional 43.5 min (95% CI: 24.9–62.1; *p* < 0.001). Tumor diameter was also independently associated with operative time, with each 1-cm increase resulting in a 6.1-minute prolongation (95% CI: 2.9–9.4; *p* < 0.001). No other variables demonstrated a significant independent effect (all *p* > 0.05) (Table 4).

**Table 4:** Multivariable linear regression analysis identifying independent predictors of operative time in adrenalectomy

Variable	B coefficient	*P* value	95% CI for B	
			Lower Bound	Upper Bound
Constant	58.77	0.041	2.48	115.1
Gender (Male vs. Female)	17.08	0.080	−2.07	36.24
Age (years)	−0.44	0.246	−1.18	0.3
BMI (kg/m²)	1.09	0.155	−0.41	2.59
Laterality (Right + Bilateral vs. Left)	13.89	0.107	−3.01	30.79
Surgical Approach (RA vs. LA)	43.51	**< 0.001**	24.86	62.15
Pathological Diameter (cm)	6.12	**< 0.001**	2.86	9.37

CI: Confidence Interval, LA: Laparoscopic adrenalectomy, RA: Robotic adrenalectomy, BMI: Body mass index (kg/m2)

Bold values indicate statistically significant differences (*p* 0.05).

## Discussion

In our cohort, the rates of intraoperative and postoperative complications did not differ significantly between the RA and LA groups, suggesting that both techniques can be performed with comparable safety in experienced hands. This finding is consistent with the results reported by Vatansever et al. who likewise demonstrated similar complication profiles for the two approaches [13]. Furthermore, a single-center study involving more than 300 patients who underwent RA identified a history of mesocolic or retroperitoneal surgery as a significant risk factor for intraoperative complications [14]. In our study, however, prior abdominal surgery was evenly distributed between groups and did not appear to influence the incidence of complications, further supporting the perioperative safety of the robotic approach in this cohort.

In the literature, the highest conversion rate for minimally invasive adrenalectomy was reported by Morino et al. in the first randomized controlled trial comparing robotic and laparoscopic adrenalectomy [15]. However, subsequent evidence has consistently demonstrated lower conversion rates with robotic platforms. A recent meta-analysis reported conversion-to-opening rates of 1.05% in the robotic group compared with 3.7% in the laparoscopic group, suggesting a potential technical benefit of the robotic approach in selected, technically demanding cases [16]. In our study, the conversion-to-opening rates were 3.2% for LA and 3.6% for RA. Obesity is another challenging situation for adrenal surgery because of the limited operative space it creates. Increasing data from recent studies suggest that the robotic platform may offer certain advantages over laparoscopy, particularly in patients with elevated BMIs [17]. In our cohort, which included one of the highest mean BMI values reported among the robotic adrenalectomy series (mean BMI: 31.68), the conversion rates remained low and comparable between the RA and LA groups. These findings imply that the robotic system may help mitigate certain technical difficulties associated with higher BMI, contributing to stable operative performance even in anatomically complex patients.

The Transfusion requirements are similar between the RA and LA, but the robotic platform may facilitate more precise dissection and hemostasis owing to enhanced magnification and improved instrument articulation [18]. A noteworthy technical aspect of our series is that all robotic procedures were performed with a Maryland bipolar dissector in combination with monopolar scissors, enabling highly controlled, fine-precision dissection and reliable hemostasis throughout the operation. Importantly, no 30-day mortality occurred in either group. This finding aligns with a meta-analysis of 17 studies reporting a 30-day mortality of 0% (0/582) for robotic adrenalectomy and 0.6% (8/1032) for laparoscopic adrenalectomy, with no significant difference between techniques [16]. Taken together, these observations reinforce the safety of robotic adrenalectomy in high-volume centers performing minimally invasive adrenal surgery.

Among the most frequently cited limitations of robotic adrenalectomy are its tendency toward longer operative times. This prolongation is multifactorial and may reflect docking requirements, team coordination, and the learning curve associated with both the surgeon and operating room staff—factors that extend the total operative time independent of the actual console dissection time [19]. In line with previous reports of longer operative times for RA compared with LA [20], our analysis showed a significantly prolonged operative time in the RA group.

A recent meta-analysis reported comparable operative times between the two techniques; however, the authors highlighted that heterogeneity in surgical protocols and the lack of side-specific analyses limited the strength of these conclusions [16]. Notably, Piccoli et al. [21]. were the first to evaluate operative duration separately for right- and left-sided adrenalectomy, demonstrating that both laterality and tumor size influence operative time—specifically, right-sided RA was significantly longer than right-sided LA was, while no difference was observed on the left. In contrast, our study revealed that RA required significantly longer operative times for both right- and left-sided procedures, suggesting that the time-related disadvantage of robotics may persist regardless of adrenal laterality. Prior evidence also supports the influence of experience and patient factors on operative time. Agcaoglu et al. [22]. reported a marked reduction in operative time after the first 10 robotic procedures, underscoring the effect of the learning curve. Additional studies have shown that tumor size and BMI may prolong the operative time; Kim et al. [23]. found shorter operative times for LA in tumors ≤ 5.5 cm, and Loy et al. [24]. demonstrated BMI-related prolongation in both RA and LA patients. However, in our multivariable linear regression analysis, operative time was independent of BMI and laterality, with only the surgical approach (RA vs. LA) and tumor diameter identified as significant predictors. These results suggest that operative time differences between the RA and LA were driven by the choice of surgical approach and tumor diameter, which are independent of BMI or laterality.

Length of hospital stay in adrenal surgery is influenced by several clinical factors, including tumor functionality—particularly in pheochromocytoma—and patient comorbidities, both of which may confound comparisons between surgical techniques. In the present cohort, however, these variables were similarly distributed between the RA and LA groups, minimizing their potential impact on postoperative recovery time. Consistent with prior studies reporting shorter hospitalization after robotic adrenalectomy, our cohort likewise demonstrated a significantly shorter length of stay in the RA group than in the LA group [8, 25, 26]. When laterality was considered, left-sided adrenalectomy was associated with a shorter hospital stay in the robotic group, whereas no significant difference was noted for right-sided procedures.

An important strength of this study is its structured, laterality-based analysis, which provides a more detailed evaluation of minimally invasive adrenalectomy than is typically presented in the literature. The right and left adrenal glands lie within distinct anatomical regions and require different technical maneuvers, making side-specific assessment clinically meaningful. Our findings showed that, despite the long-held assumption that laterality may influence perioperative risk, the complication and transfusion rates were comparable between right- and left-sided procedures. Notably, the operative time remained consistently longer for RAs on both sides, suggesting that the time-related disadvantage of the robotic approach persists irrespective of adrenal laterality. By offering one of the few comparative assessments stratified by tumor side, this study adds clarity to an understudied dimension of adrenal surgery and highlights the value of further side-stratified investigations in future research. However, this study has several limitations. First, it represents a single-center, nonrandomized, retrospective analysis, which may introduce inherent selection bias—particularly because early cases may have been preferentially assigned to one technique depending on surgeon experience, resource availability, or clinical judgment. Second, although the sample size is comparable to that of many published series, it remains relatively modest and may limit the strength of subgroup comparisons. Third, cost-effectiveness—one of the most frequently debated disadvantages of robotic adrenalectomy—was not evaluated in this study. Previous studies have often reported higher overall costs associated with RA [27]; however, more recent analyses incorporating operating room resource utilization, hospital stay, and perioperative consumables have demonstrated increasingly comparable cost profiles between the two approaches [28, 29]. Additionally, although all procedures were performed in a high-volume endocrine surgery center, the learning curve—particularly during the early phase of robotic adrenalectomy—may have influenced operative time and perioperative outcomes. Given these considerations, multicenter, prospective, randomized studies with comprehensive economic assessments are needed to validate our findings and further clarify the clinical and financial impact of robotic adrenalectomy in high-volume endocrine surgery settings.

## Conclusion

Robotic adrenalectomy can be performed safely and achieves perioperative outcomes comparable to those of laparoscopic adrenalectomy in experienced centers. In our cohort, RA was associated with a shorter hospital stay but a longer operative time, whereas complication, transfusion, and conversion rates were similar between the two approaches. Side-specific analysis revealed that the time-related disadvantage of RA persisted regardless of adrenal laterality. Overall, these findings add to the growing body of evidence suggesting that RA may represent a feasible and ergonomically favorable option for minimally invasive adrenal surgery in selected patients, particularly when performed in high-volume endocrine surgery centers.

## Data Availability

The datasets generated and/or analyzed during the current study are not publicly available owing to institutional regulations but are available from the corresponding author upon reasonable request.
